# iPSC-derived MSC therapy induces immune tolerance and supports long-term graft survival in mouse orthotopic tracheal transplants

**DOI:** 10.1186/s13287-019-1397-4

**Published:** 2019-09-23

**Authors:** Mohammad Afzal Khan, Fatimah Alanazi, Hala Abdalrahman Ahmed, Talal Shamma, Kilian Kelly, Mohamed A. Hammad, Abdullah O. Alawad, Abdullah Mohammed Assiri, Dieter Clemens Broering

**Affiliations:** 1 Organ Transplant Research Section, King Faisal Specialist Hospital and Research Centre, Riyadh, Kingdom of Saudi Arabia; 2 Comparative Medicine Department, King Faisal Specialist Hospital and Research Centre, Riyadh, Kingdom of Saudi Arabia; 3 Cynata Therapeutics Limited, Melbourne, Australia; 4 National Center for Stem Cell Technology, King Abdulaziz City for Science and Technology, Riyadh, Saudi Arabia; 5 College of Medicine, Alfaisal University, Riyadh, Saudi Arabia; 6 Institute for Research and Medical Consultations, Imam Abdulrahman Bin Faisal University, Dammam, Saudi Arabia

**Keywords:** Stem cell therapy, Immunotolerance, Regulatory T cells, Hypoxia and ischemia, Microvasculature, Orthotopic tracheal transplants

## Abstract

**Background:**

Lung transplantation is a life-saving surgical replacement of diseased lungs in patients with end-stage respiratory malfunctions. Despite remarkable short-term recovery, long-term lung survival continues to face several major challenges, including chronic rejection and severe toxic side effects due to global immunosuppression. Stem cell-based immunotherapy has been recognized as a crucial immunoregulatory regimen in various preclinical and clinical studies. Despite initial therapeutic outcomes, conventional stem cells face key limitations. The novel Cymerus™ manufacturing facilitates production of a virtually limitless supply of consistent human induced pluripotent stem cell (iPSC)-derived mesenchymal stem cells, which could play a key role in selective immunosuppression and graft repair during rejection.

**Methods:**

Here, we demonstrated the impact of iPSC-derived human MSCs on the development of immune tolerance and long-term graft survival in mouse orthotopic airway allografts. BALB/c → C57BL/6 allografts were reconstituted with iPSC-derived MSCs (2 million/transplant/at d0), and allografts were examined for regulatory T cells (Tregs), oxygenation, microvascular blood flow, airway epithelium, and collagen deposition during rejection.

**Results:**

We demonstrated that iPSC-derived MSC treatment leads to significant increases in hTSG-6 protein, followed by an upregulation of mouse Tregs and IL-5, IL-10, and IL-15 cytokines, which augments graft microvascular blood flow and oxygenation, and thereby maintained a healthy airway epithelium and prevented the subepithelial deposition of collagen at d90 post transplantation.

**Conclusions:**

Collectively, these data confirmed that iPSC-derived MSC-mediated immunosuppression has potential to establish immune tolerance and rescue allograft from sustained hypoxic/ischemic phase, and subsequently limits long-term airway epithelial injury and collagen progression, which therapeutically warrant a study of Cymerus iPSC-derived MSCs as a potential management option for immunosuppression in transplant recipients.

## Background

Lung transplantation is a life-saving surgical procedure for patients with end-stage lung diseases. Unfortunately, this therapeutic strategy is vulnerable by the occurrence of chronic rejection, which occurs when the recipient’s immune response impairs the transplanted organ through microvascular disruption. Loss of graft microvasculature has been recognized as an underlying cause of chronic rejection, which is manifested by terminal airway fibrosis of the transplanted organs [1, 2]. Existing immunosuppressive drugs for organ transplantation may achieve sufficient immunosuppression to prevent organ rejection or limit autoreactivity, but they are typically not successful in achieving long-term survival of the graft or preventing progression of fibrosis and chronic rejection [3]. Existing immunosuppressive drugs are also associated with low specificity leading to toxicity and increased risk of infections and malignancies.

Immunotolerance is a major immunological phase, which facilitates reparative microenvironment during transplantation, and plays a vital role to suppress alloimmune responses. A number of therapeutic options have been investigated to increase the number of regulatory T cells (Tregs) during allograft rejection, for example, through C5a blockade, stem cell therapy, and immune checkpoints, which potentially suppress infiltration of CD4^+^ T cells and antibody-mediated complement activation on vascular endothelial cells [1, 2, 4–8]. Cell-mediated immunotherapy is emerging as an alternative in single and combination therapies to rescue rejecting transplants. In particular, mesenchymal stem cells (MSCs) are multipotent self-renewing precursor cells that are found mainly in the bone marrow and also reported in other tissues, such as the umbilical cord blood and muscle and adipose tissue [9, 10]. Functionally, MSCs have enormous potential for alleviating the pathological state of disease by the release of various bioactive molecules such as cytokines, chemokines, and growth factors, in addition to their immunosuppressive and immunoregulatory properties; therefore, there has been growing therapeutic interest of MSCs as an option for many incurable diseases [11]. MSCs have been tested in animal models and clinical trials for the treatment of numerous diseases including transplantation [12–18]. A growing interest in the area of transplantation medicine, led by an improved understanding of the role of MSCs in immunosuppression, tissue repair, and fibrosis, has seen recent focused efforts to explore their potential in the active management of transplantation [17, 19–21].

A number of preclinical and clinical studies have been conducted to assess the effects of donor-derived MSCs on the outcome of solid organ transplants, with generally encouraging results, including reductions in the incidence of graft rejection, improved patient survival, and reduced requirements for immunosuppressive drugs [17, 21, 22]. However, there remains a need for more extensive preclinical studies and further investigation of how these cells affect various pathological and cellular compartment post treatment. Furthermore, production of MSC-based therapies by conventional means is associated with significant limitations with respect to reproducibility and scalability. Conventional methods of MSC production rely on isolating cells from tissue donations, followed by culture expansion. However, as a result of research conducted by numerous independent groups, it is now widely accepted that extensive culture expansion of donor-derived MSCs leads to loss of potency and senescence [23–26]. Furthermore, significant donor to donor variability has been observed [24, 27, 28], while recruitment of donors is time-consuming, costly, and fraught with risk. Consequently, this problem cannot be solved by recruiting more donors and lowering expansion levels. Importantly, these limitations have been borne out by the clinical trial data available to date, which strongly indicate better outcomes with minimally expanded BM-MSCs than with extensively expanded donor-derived MSCs [23, 24].

Cynata is developing an alternative approach to producing MSCs (the Cymerus™ process), which is intended to avoid the limitations described above. The Cymerus™ process involves producing cell-based mesenchymoangioblasts (MCAs), which are originally derived from induced pluripotent stem cells (iPSCs). iPSCs, which can be produced by reprogramming adult cells using non-integrating episomal methods [29], have an effectively infinite capacity to reproduce themselves without loss of their key characteristics [30], in addition to the ability to differentiate into any other type of adult cell. Consequently, by harnessing the expansion capacity of iPSCs, an effectively limitless number of Cymerus iPSC-derived MSC doses can be produced from a single iPSC bank, without the need to excessively expand the MSCs after differentiation.

Previous studies in mice have demonstrated therapeutic benefits of Cymerus iPSC-derived MSCs in mouse models of hind limb ischemia [31], chronic allergic airways disease [32], and severe acute graft versus host disease (GvHD; unpublished data). A clinical trial is also underway to test the clinical efficacy of Cymerus iPSC-derived MSCs (CYP-001) in human patients with steroid-resistant acute GvHD (ClinicalTrials.gov Identifier: NCT02923375). The previously published studies showed that Cymerus iPSC-derived MSCs increased blood flow, reduced necrosis and maintained muscle mass after induction of hind limb ischemia, and suppressed inflammation and airway hyperresponsiveness and reversed airway fibrosis during chronic allergic airways disease. However, the effect of Cymerus iPSC-derived MSCs on cellular immunity, especially on the balance of T regulatory cells and T effector cells, had not been investigated.

We investigated the possible therapeutic benefits of Cymerus iPSC-derived MSCs on immune tolerance, graft hypoxia, ischemia, tissue injury, and progression of fibrosis during airway transplantation. In order to evaluate iPSC-derived MSC-mediated therapeutic intervention, we adopted a well-established mouse model of orthotopic trachea transplantation, which easily replicates microvascular changes and real-time monitoring of blood flow during rejection [1, 4, 33]. The key objectives were to investigate if therapeutic administration of iPSC-derived MSCs may increase regulatory T cells in allografts, promote microvascular tissue repair, limit fibrosis, and support the long-term graft survival. We believe that targeting iPSC-derived MSC-mediated T regulatory cell and T effector cell stoichiometry during rejection episodes may be a novel and effective therapeutic strategy in combination with other immunosuppressive regimens for preventing the development of chronic rejection.

## Methods

### Mice

All MHC-mismatched mice selected for this investigation were acquired from the Jackson Laboratory (JAX, USA) and preserved as an original breeder colony in our King Faisal specialist Hospital and Research Centre animal facility at Riyadh, Saudi Arabia. All mice used in this study were later approved by Animal Care and Use Committee (ACUC).

#### C57BL/6

C57BL/6 (B6.H-2b) mice were selected as tracheal graft recipients in all allograft conditions while they were selected as tracheal graft donors in syngraft conditions as described in the experimental plan (Table 1).

**Table 1:** Details of graft, cell dosing, and graft assessment plan

Donor	Recipient	Treatment	Group purpose	Assessments (d)
C57BL/6	C57BL/6	Saline	Saline-treated syngeneic control	8–14, 20, 28, 90
BALB/c	C57BL/6	Saline	Saline-treated allogeneic control	8–14, 20, 28, 90
BALB/c	C57BL/6	iPSC-derived MSCs	To test the efficacy of iPSC-derived MSCs	8–14, 20, 28, 90

Sample size (*n*) = 12–18 transplants/time points/experiment

#### BALB/c

BALB/c (H-2d) mice were selected as allogeneic tracheal graft donors for the C57BL/6 mice recipients in the orthotopic trachea transplants.

#### Outline of OTT procedure

The surgical procedure of orthotopic tracheal transplant (OTT) was performed under sterile conditions, and different groups were surgically transplanted as originally planned and listed in Table 1. In brief, recipient mice were anesthetized (ketamine 100 mg/kg and xylazine 20 mg/kg) and the trachea was bisected with small incision, and donor tracheal segment of four to six rings was surgically sutured into the recipient with 10-0 nylon suture (AROSurgical, USA) at each anterior and posterior end of the bisected trachea. Next, all the surrounding tissues were placed and the overlying skin was closed with 5-0 silk suture [33, 34]. All transplanted mice were given carprofen (dose 5 mg/kg × SC) and Zolecin (dose 100 mg/kg × SC) and were monitored for any respiratory distress-stridor in the first 24 h after surgery.

To examine the transplanted allografts, syngrafts and iPSC-derived MSC-treated allografts, we selected specific days post tracheal transplantation, which starts from d8 (phase of healthy microvasculature), d9 (point of minimum donor-recipient microvascular), d10 (point of donor-recipient microvascular loss)–d14 (donor-recipient microvascular loss continued), d20–28 (some microvascular recovery), and d90 (mainly fibrotic airways) to better see the cellular and molecular effects of ongoing therapy for studying rejecting allografts [1, 4, 35].

#### iPSC-derived MSCs and dosing

iPSC-derived MSCs were produced using the Cymerus manufacturing process by Cynata Therapeutics Limited, Australia. In brief, the iPSCs were derived from CD34-enriched human peripheral blood mononuclear cells (PBMCs) using a transgene-free, viral-free, and feeder-free reprogramming procedure. After culture expansion in chemically defined conditions [36], the iPSCs were then subjected to a directed differentiation process involving the formation of precursors called mesenchymoangioblasts, which in semisolid medium form FGF2-dependent compact spheroid colonies containing mesenchymal cells [37]. The resultant MSCs were cultured in serum-free expansion medium, harvested, and then cryopreserved in cryoprotectant medium containing 10% human serum albumin and 2.5% dimethylsulfoxide (DMSO). The expression of defining MSC markers (CD73, CD90, and CD105) was confirmed by flow cytometry analysis. The cells were stored and transported in liquid nitrogen until further use. For therapeutic use, each vial of iPSC-derived MSCs was thawed and reconstituted in PBS before injection. A single intravenous injection of maximum 2 × 10^6^ cells (> 99% of purity) was slowly injected 1 day before the trachea transplantation via retro-orbital infusion [1].

### Analysis of T regulatory cells

To analyze the T lymphocytes for CD4^+^ and FOXP3^+^ expression during rejection, blood samples were collected (BD vacutainers) and lymphocyte buffy coat was separated through Hisptopaque gradient procedure as described [7, 38, 39], and mouse Treg-specific markers were stained with APC-conjugated anti-mouse CD4^+^ (Clone RM4-5 RUO, BD Pharmingen), PE-Cy7 CD25^+^ (Clone PC 61 RUO, BD Pharmingen), and PE-conjugated FOXP3^+^ (Clone MF23 RUO, BD Pharmingen) respectively as recommended by BD Pharmingen assay, which specifically flow sort CD4^+^CD25^+^FOXP3^+^ Treg subpopulation from mixed lymphocytes. Data were recorded at the flow rate of 14 μl/min, and minimum 500,000 events were collected and analyzed through BD accuri C6 integrated software [7, 39].

Further, to examine the intragraft infiltration of CD4^+^FOXP3^+^ Tregs, all control and iPSC-derived MSC-treated allografts were stained for CD4^+^ and FOXP3^+^ marker in grafts. In brief, harvested and Tissue-Tek O.C.T. medium (Sakura Finetek, Japan) processed grafts were sliced (5 μm) through A cryostat (HM550; Microm) and the sections were mounted on superfrost/plus slides (Fisher Scientific) for immunofluorescence staining as described [1, 4, 7, 39]. After methanol/acetone (1:1) fixation for 10 min at − 20 °C, the slides were washed and incubated with 10% donkey serum for 30 min. Next, slides were overnight incubated with either rat anti-mouse CD4 (BD biosciences, USA), rabbit anti-mouse FOXP3 (abcam, USA), and goat anti-human TSG-6 (R&D Systems)-specific primary antibodies. After O/N incubation, slides were washed and incubated for 1 h with Cy3 donkey anti-rat (Jackson Immuno research, USA), Alexa 647 donkey anti-rabbit (Jackson Immuno research, USA), or Alexa 488 Donkey anti-rat (Jackson Immuno research, USA) secondary antibodies. After incubation with secondary antibody, slides were thoroughly washed and fixed in vectashield mounting medium (Vector Laboratories, USA) for immunofluorescence image analysis. Immunofluorescence analysis was performed through image acquisition of five random high-powered fields per slide for a selected individual marker on EVOS FL auto cell imaging system (Life technologies, USA), and the percentage of co-localization of two markers was quantified through the mean integrated fluorescence intensity per treatment group using ImageJ program [1, 4, 40].

### Analysis of functional microvasculature

To examine the functional microvascular blood flow between donor and recipient graft, transplants were anesthetized and quickly injected intravenously with 100 μl (1 mg/ml FITC-conjugated *Lycopersicon esculentum*) of tomato lectin [40] and wait for 5 min before flushing the whole vasculature with 1% PFA (paraformaldehyde) via the aorta. After PFA washing, graft was harvested and fixed in 1% PFA at 4 °C for 10 min. An immunofluorescence microscope (EVOS FL auto cell imaging system, Life technologies, USA) was used to examine the pattern and density of graft microvasculature, and morphometric analysis of perfused microvasculature was quantified by capturing five random high-powered fields per slide, and the mean integrated fluorescent intensity was calculated through ImageJ program [1, 4].

### Analysis of graft oxygenation and microvascular blood flow

The oxygen (tpO_2_ mmHg) and blood perfusion (BPUs) during rejection were measured by combined oxygen and blood flow sensors (model NX-BF/OF/E, Oxford Optronix, UK) as described earlier with some modifications [1, 4, 7, 33]. Briefly, the transplanted mouse was anesthetized and the graft was exposed for oxygen and blood flow measurement. Next, a 23G needle was used to make a hole in the graft, and a combined sensor was gradually inserted through a micromanipulator until it reached the epithelium of the graft. Since the graft is orthotopic, it always remains in the vicinity of inhaled oxygen, which may interfere with tissue oxygen. To avoid and minimize this inhaled oxygen, the sensor was lowered until the tpO_2_ levels decrease to 5 mmHg or less (indicating a zeroing effect induced by tissue compression), and subsequently raised in small increments through micromanipulator until the tpO_2_ and BPU reading plateaus and a consistent reading was obtained [33, 34]. Of note, detachment of the sensor from the tissue epithelium spiked the oxygen content (> 45 mmHg); therefore, we routinely optimized this measurement while the sensor remained in firm contact with the epithelium, which we regulate through micromanipulator.

### Analysis of airway epithelium and collagen deposition

Pathological changes in airway epithelium and collagen in iPSC-derived MSC-treated and MSC-untreated control allografts were evaluated by H&E and trichrome staining as described [7, 39, 41]. In brief, harvested and Tissue-Tek O.C.T. medium (Sakura Finetek, Japan)-processed graft sections on superfrost/plus slides (Fisher Scientific) were stained by H&E and trichrome to detect any pathological and structural perturbations in the airway epithelium and collagen. Image acquisition and morphometric assessments of collagen deposition (blue band) were quantified by capturing five random high-powered fields per slide, and the pixel size was converted into micrometers to calculate mean band width (μm^2^) per treatment group using ImageJ program [4, 42].

### Analysis of serum cytokines

Quantitative analysis of serum levels of proinflammatory and anti-inflammatory cytokines was performed by Milliplex MAP Mouse Th17 Magnetic Bead (Cat # MTH17MAG-47 K). Briefly, serum was separated from the blood after spinning at 1200 RCF for 10 min and stored at − 80 °C for further use. Quantitative estimation of serum cytokines was performed as suggested by the manufacturer-approved protocol through antibody-linked magnetic beads on a 96-well plate. Plates were washed twice with wash buffer and incubated at room temperature with biotinylated detection antibodies, and streptavidin-PE was added for 30 min with the shaking incubator. Next, plates were washed and recorded on Luminex 200 instrument in duplicates.

### Data analysis

Statistical comparison between various groups was performed using two-way ANOVA with Bonferroni multiple comparisons for post hoc analyses, whereas differences between single time points were compared by one-way ANOVA on GraphPad prism software, and a *p* value < 0.05 was considered significant.

## Results

### iPSC-derived MSC therapy is associated with human TSG-6 and peripheral Treg increase

**Fig. 1:**
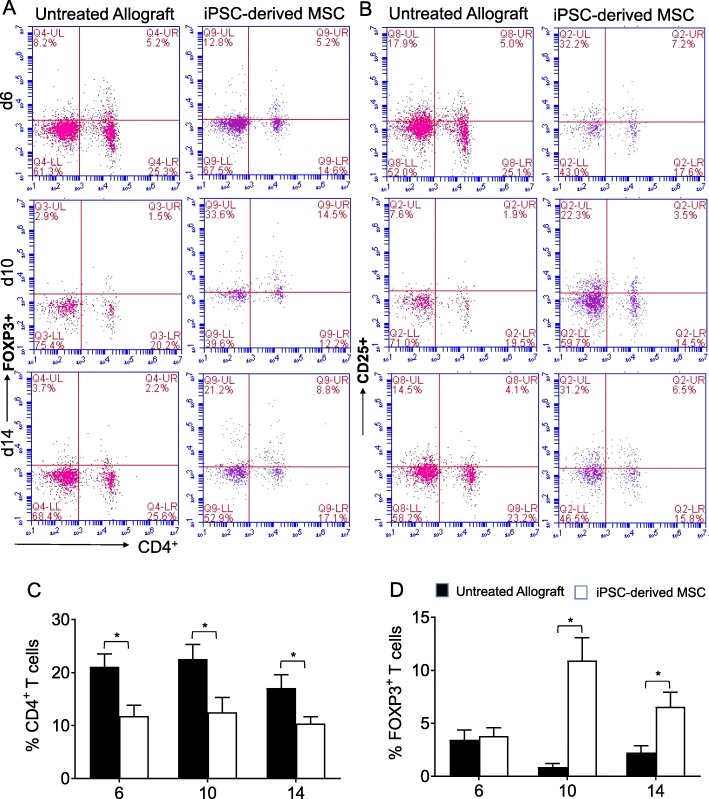
iPSC-derived MSC therapy is associated with increased CD4^+^CD25^+^FOXP3^+^ Treg in the peripheral blood. Flow cytometry analysis of Tregs from the peripheral blood of allograft recipients on d6, d10, and d14 post transplantation. **a**, **b** Flow cytometric analysis of CD4^+^CD25^+^FOXP3^+^ lymphocytes in the peripheral blood. **c** Percentage of CD4^+^ T cells in a gated lymphocyte population. **d** Percentage of FOXP3^+^ T cells in gated lymphocyte population. Data are presented as means with SE of 18 transplants/time point/experiment. *p < 0.05

The anti-inflammatory and immunosuppressive properties of stem cell have been well reported, which modulate innate and adaptive immune responses and modulate antigen-specific T cell receptor (TCR), Notch signaling, and FOXP3 stability for Treg clonal expansion [43–46]. Here, we tested the impact of adoptive transfer of iPSC-derived human MSC therapy on secreted hTSG-6 protein in injured transplants, and its cellular and molecular effects on peripheral and intragraft infiltration of Tregs during airway allograft rejection. To investigate the roles of iPSC-derived MSC therapy and secreted hTSG-6 proteins during airway allograft rejection, we surgically transplanted MHC-incompatible C57BL/6 (B6, H-2^b^) with tracheas of BALB/c (H-2^d^) donors. Next, blood lymphocytes were isolated from untreated BALB/c (H-2^d^) → C57BL/6 (H-2^b^) control allografts and iPSC-derived MSC-treated BALB/c (H-2^d^) → C57BL/6 (H-2^b^) allografts for peripheral CD4^+^CD25^+^FOXP3^+^ Treg counting at d6, d10, and d14 post transplantation. We found that allografts that were accompanied by iPSC-derived MSC therapy exhibited a remarkable increase in CD4^+^CD25^+^FOXP3^+^ Tregs in the peripheral blood, compared to untreated control allografts at d6, d10, and d14 post transplantation (Fig. 1a–d). These findings indicate that iPSC-derived MSC therapy has potential to establish an immunologically favorable phase of immune tolerance through the peripheral CD4^+^CD25^+^FOXP3^+^ Tregs, which is crucial to favor immunosuppressive environment in systemic circulation as well as inside the graft, which is required for regulatory and reparative phase during rejection.

**Fig. 2:**
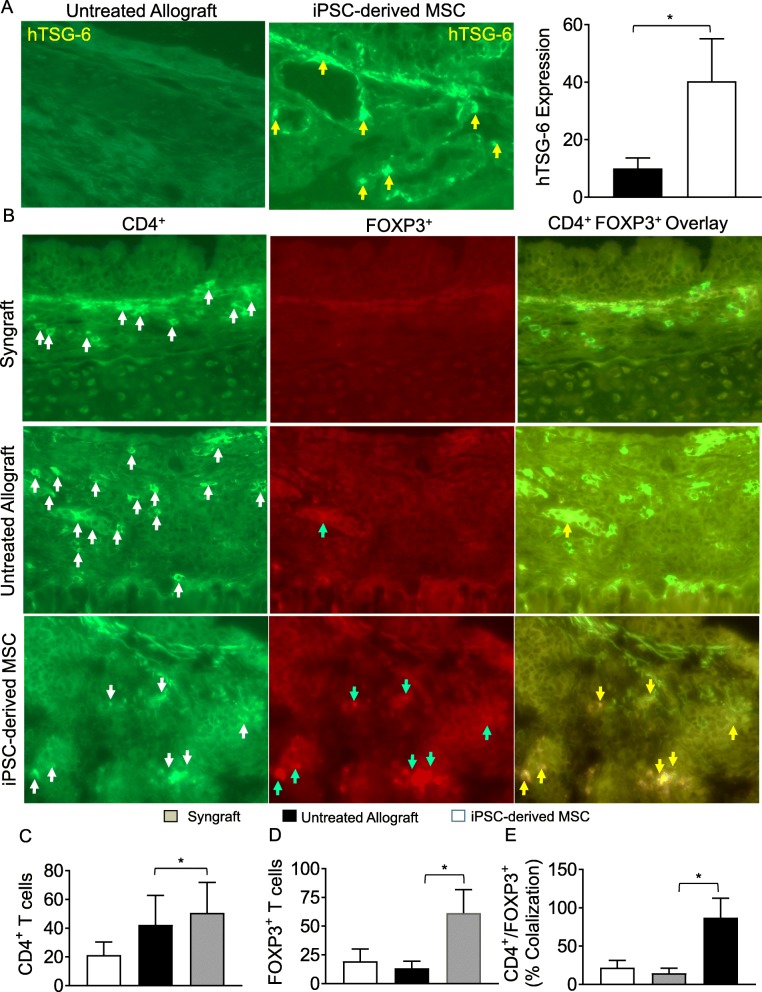
iPSC-derived MSC therapy increased infiltration of Treg in allograft. **a** Immunofluorescent staining for subepithelial human TSG-6 expression at d10 post transplantation. **b** Immunofluorescent staining for CD4^+^FOXP3^+^ lymphocytes at d10 post transplantation. **c**–**e** Morphometric analysis of graft infiltrating CD4^+^ T cells, FOXP3^+^ cells, and CD4^+^FOXP3^+^ co-localization (Tregs). Arrows highlighted individual CD4^+^ T cells (white arrows), FOXP3^+^ cells (blue arrows), and CD4^+^FOXP3^+^ Treg cells (yellow arrows). Data are presented as means with SE of 12 transplants/time point/experiments. *p < 0.05. Original magnification, × 40

To investigate the intragraft infiltration of peripheral CD4^+^CD25^+^FOXP3^+^ Tregs, we next tested whether the observed systemic increase in Tregs is also associated with a subepithelial deposition of hTSG-6 proteins, Tregs, or suppression in number of CD4^+^ T cells in allograft. We immunostained iPSC-derived MSC-treated allografts for TSG-6, CD4, and FOXP3 at d10 post transplantation and analyzed through immunofluorescence imaging. Image morphometric analysis showed an increase in hTSG-6 protein expression and subepithelial FOXP3, CD4^+^ T cells overlay in iPSC-derived MSC-treated allografts compared to untreated control allografts at d10 post transplantation (Fig. 2a–e). Based on the findings, the MSC-derived iPSC therapy is associated with an increase in subepithelial hTSG-6 expression followed by a systemic and intragraft increase in CD4^+^FOXP3^+^ Tregs, which will support the immune tolerance phase through regulatory signals for microvascular restoration and tissue repair post therapy [39, 47].

### iPSC-derived MSC therapy reinstates graft microvasculature and improves graft oxygenation and microvascular blood flow

**Fig. 3:**
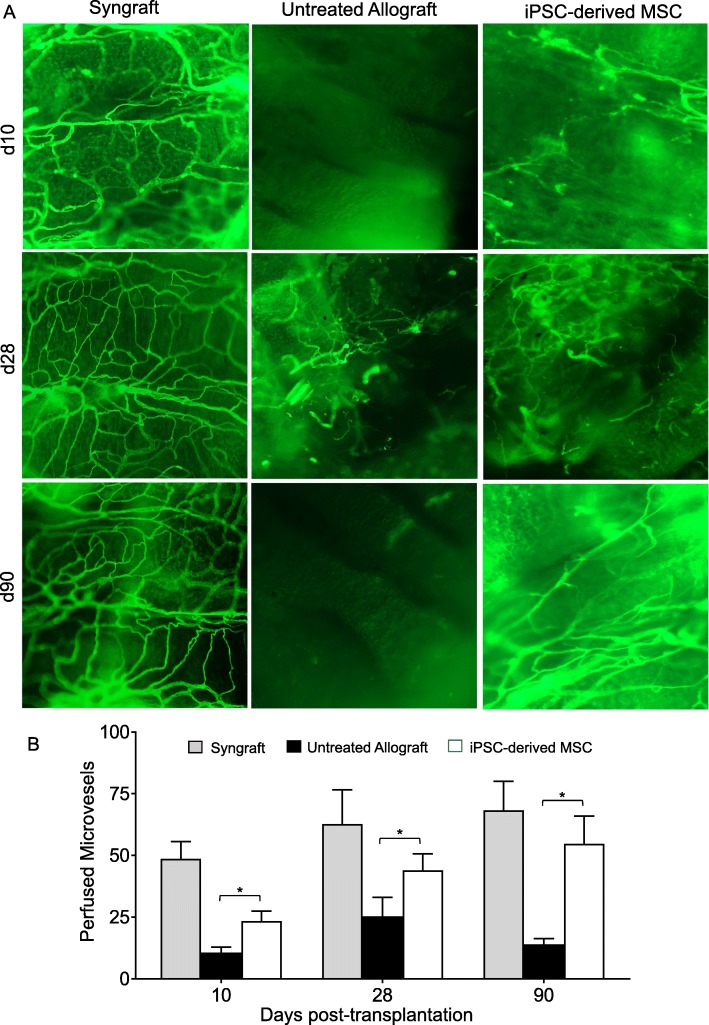
iPSC-derived MSC therapy preserves graft functional microvasculature. **a** Lectin binding assay of whole-mount tracheal grafts on d10, d28, and d90. Original magnification, × 20. **b** Morphometric assessment of perfused vasculature (Lectin-stained vessels/unit area) in allo-transplanted groups at different time points. Data are presented as means with SE of 12 transplants/time point/experiment. *p < 0.05

While the effects of stem cell therapies on airway inflammation, vascularization, angiogenesis, and capillary formation had previously been reported [48–50], here we further delineated the effects of Cymerus iPSC-derived MSCs and surprisingly found an increase in Tregs in iPSC-derived MSC-treated allografts. Of note, these findings are consistent with the fact that, in the current OTT transplantation model, CD4^+^FOXP3^+^ Tregs-mediated immune tolerance is associated with microvascular tissue repair [39, 47]. To evaluate the reparative effects of iPSC-derived MSC cells on donor-recipient graft microvascular connections, and oxygenation state of allografts during rejection, here, we further tested the hypothesis that iPSC-derived MSC-mediated Treg immune tolerance would reestablish microvascular connections between donor-recipient grafts, and would thereby revive microvascular blood flow and tissue oxygenation. To investigate this, iPSC-derived MSC reconstituted and untreated control allografts were monitored for tissue oxygenation, microvascular blood perfusion, and donor-recipient microvasculature blood flow in rejecting allografts. Next, we transplanted MHC-incompatible BALB/c (H-2^d^) → C57BL/6 (H-2^b^) allografts, BALB/c (H-2^d^) → C57BL/6 (H-2^b^) iPSC-derived MSC-treated allografts, and MHC compatible C57BL/6 (H-2^b^) → C57BL/6 (H-2^b^) syngrafts. Of note, to investigate microvascular reestablishment, all untreated control and iPSC-derived MSC-treated allografts were examined by FITC-lectin binding assay, which specifically detects the pattern of donor-recipient functional microvasculature during rejection [1, 4, 51]. We found that donor-recipient graft microvasculature in syngrafts remained connected with high vascular density throughout the period of 4 weeks of transplantation. However, iPSC-derived MSC-treated allografts showed improved microvascular connection at d10, d28, and d90 as compared to untreated control allografts, which lost microvascular flow and remained poorly oxygenated with significantly lower vascular density (Fig. 3a, b). These findings support the notion that iPSC-derived MSC treatment has the potential to promote the microvascular flow between donor and recipient graft during rejection.

**Fig. 4:**
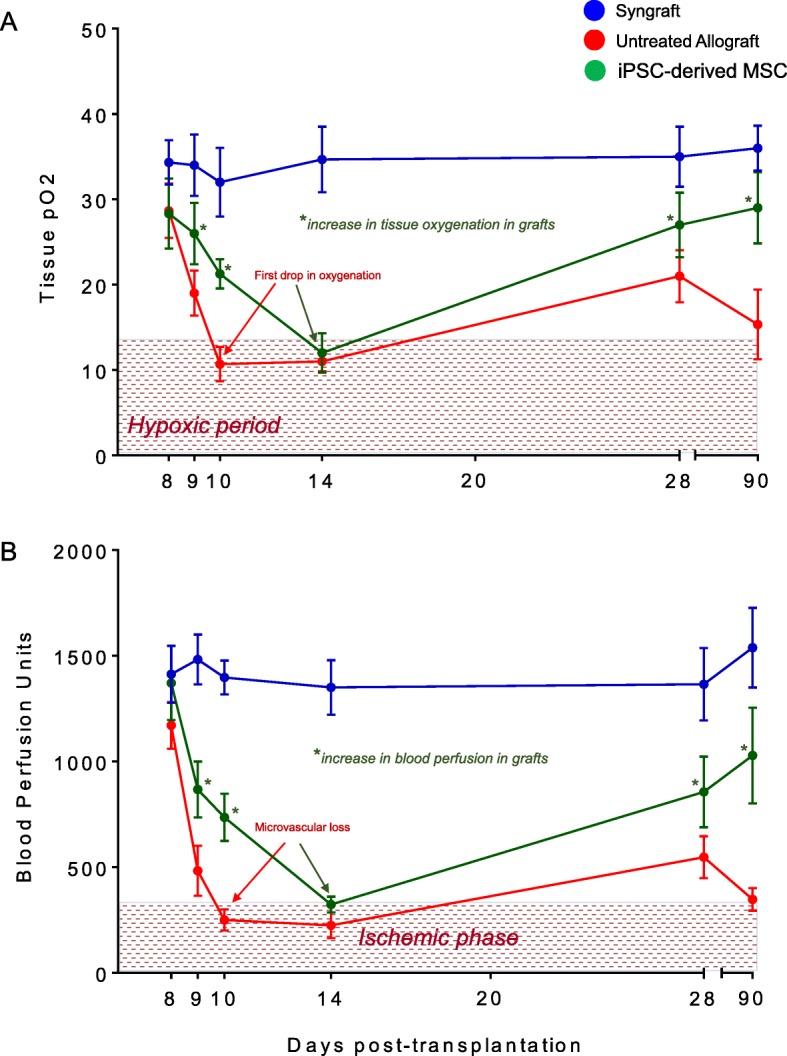
iPSC-derived MSC therapy-mediated Treg induction improves tissue pO_2_ and blood perfusion. **a** Tissue pO_2_ (mean ± SE, mmHg) and **b** blood perfusion (mean ± SE, units) were plotted over different time points. Data are presented as means with SE of 18 transplants/time point/experiment. *p < 0.05

Furthermore, these findings support the hypothesis that revival of microvascular connections between donor-recipient graft in iPSC-derived MSC-treated allografts may be vital for maintaining high oxygenation and perfused microvascular state. To test this, we examined the levels of tissue oxygenation and microvascular blood flow (measured in blood perfusion units, BPUs) in syngrafts, untreated control allografts, and iPSC-derived MSC-treated allografts from d4–d28 post transplantation (Fig. 4a, b). We found that all transplants of untreated control allografts, iPSC-derived MSC-treated allografts, and the syngraft group showed a significant increase (*p* < 0.05) in tissue oxygenation and blood perfusion after the first microvascular hook-up at d4 and remained oxygenated until d8 post transplantation without any sign of donor-recipient microvascular rejection and associated graft ischemia (Fig. 4a, b). The data also highlighted that untreated control allografts passed through a prolonged phase (d9–d14) of graft hypoxia and ischemia and only showed a delayed but inadequate revival in tissue oxygenation and blood microvascular flow by d28 post transplantation (Fig. 4a, b). These findings might have been related to the fact that massive alloimmune inflammatory environment in untreated allografts accelerated microvascular tissue injuries and delayed tissue hypoxic and ischemic state, which might have resulted in graft rejection.

Next, we explored the protective effects of the adoptive transfer of iPSC-derived MSCs in allografts, and our initial findings demonstrated a significantly reduced overall hypoxic and ischemic phase, and treated allografts remained significantly oxygenated and vascularized at d9–d13 and only showed a brief phase of hypoxia/ischemia around d14 followed by a significant and rapid rise of tissue oxygenation and blood perfusion until d20–d28 post transplantation (Fig. 4a, b). In summary, syngrafts enjoyed a consistent long phase of tissue oxygenation and perfusion from d4 to d28 without any sign of microvascular loss but rejecting allografts passed through a comparatively longer hypoxic/ischemic state from d10 to d14, while iPSC-derived MSC-treated allografts showed only a brief phase of hypoxic/ischemic state at d14 and enjoyed relatively longer phase of graft oxygenation/perfusion from d4 to d13 (Fig. 4a, b). This brief hypoxic and ischemic phase in iPSC-derived MSC-treated allografts followed by a rapid rise in oxygenation and blood perfusion compared to untreated allografts supports the notion that iPSC-derived MSC treatment triggers an immunoregulatory and immunosuppressive environment, which favors microvascular-associated repairs (Fig. 4a, b). These findings further indicate that iPSC-derived MSC delays the onset of acute rejection and thereby shortens the phase of hypoxia/ischemia as seen in untreated allografts. (Fig. 4a–d). Collectively, our data highlighted a potential therapeutic effect of iPSC-derived MSCs in microvascular graft repair during rejection.

### iPSC-derived MSC therapy attenuates inflammatory cytokines while promotes regulatory cytokines

**Fig. 5:**
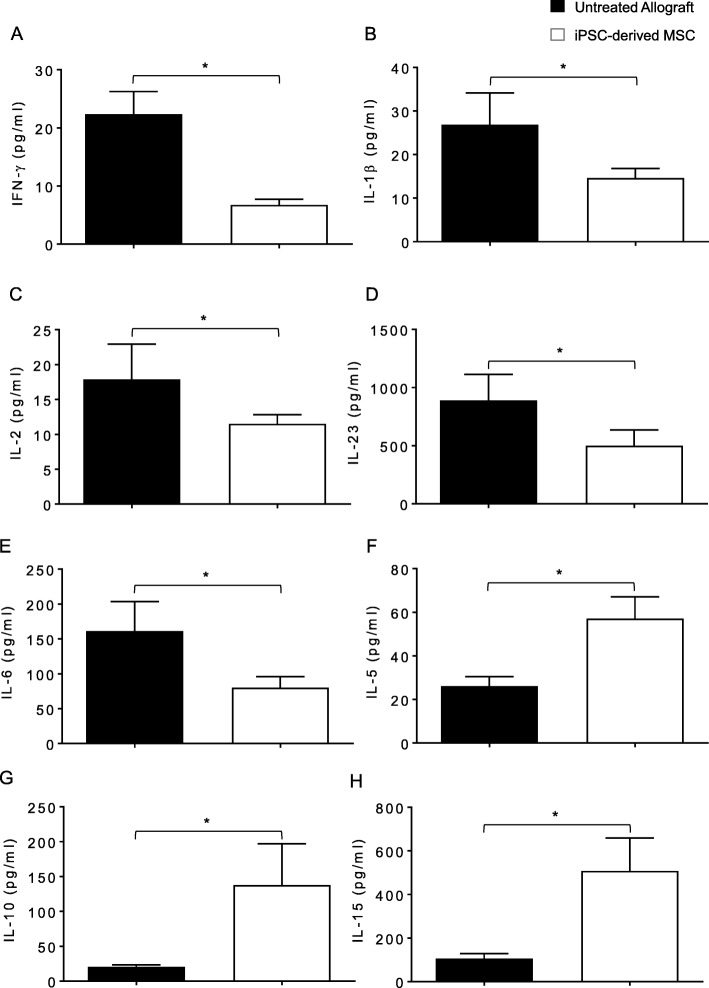
iPSC-derived MSC therapy promotes regulatory cytokines and anti-inflammatory cytokine. **a**–**h** Quantitative analysis of serum cytokines (pg/ml) shows an increase in regulatory cytokines (IL-5, IL-10, IL-15) while a decrease in inflammatory cytokines (IFN-γ, IL-1β, IL-2, IL-23, IL-6) at d10 post transplantation. Data are presented as means with SE of 18 transplants/time point/experiment. *p < 0.05

To investigate that the immunosuppressive and regulatory effects of iPSC-derived MSCs on microvascular-associated graft repairs, we quantified the serum cytokine in iPSC-derived MSC-treated and MSC-untreated control allografts. Our ELISA analysis demonstrated that iPSC-derived MSC-treated allografts showed a significant decrease in serum levels of IFN-γ, IL-1β, IL-2, IL-6, and IL-23 proinflammatory cytokines while significantly upregulated serum levels of IL-5, IL-10, and IL-15 regulatory mediators compared to untreated control allografts (Fig. 5a–h). Thus, the presence of peripheral Tregs in iPSC-derived MSC treatment allografts potentially established an immunoregulatory phase through the upregulation of serum IL-10/IL-5/IL-15 levels, thereby checking inflammatory IFN-γ, IL-1β, IL-2, IL-6, and IL-23 cytokines during rejection. The cytokine profile revealed that iPSC-derived MSC-mediated immunosuppression established a regulatory immune-milieu which most likely affects the tissue-associated microvascular repair during transplantation. Collectively, these findings indicate that microvascular recovery in iPSC-derived MSC-treated allografts initiated due to a turning on of the anti-inflammatory and regulatory microenvironment, which favors microvascular associated repair of rejecting transplant.

### iPSC-derived MSC therapy limits airway epithelial injury and prevents collagen deposition

**Fig. 6:**
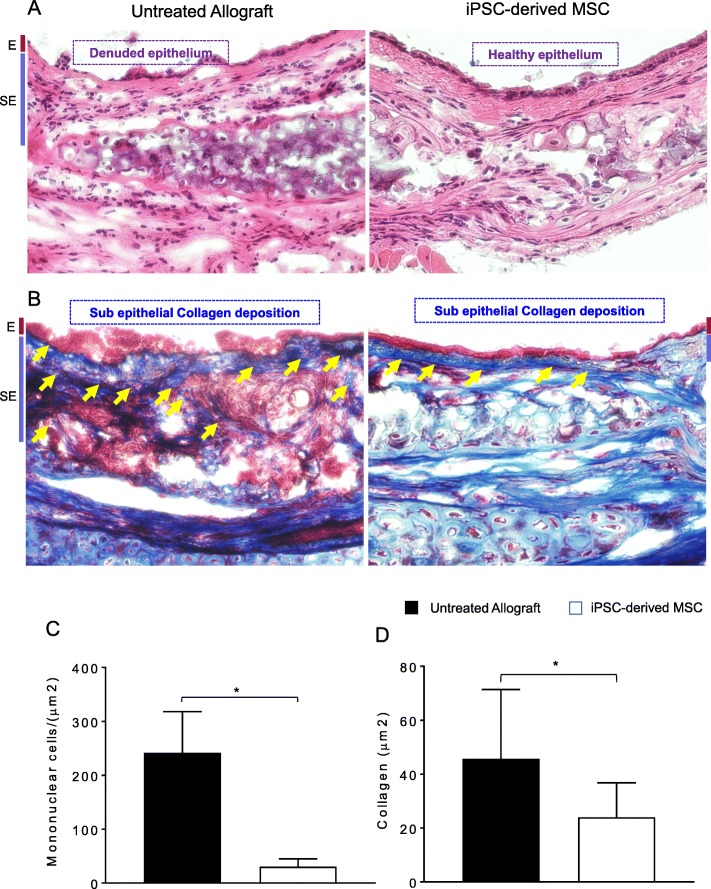
iPSC-derived MSC therapy improves allograft health and prevents collagen deposition. **a**, **c** H&E staining and subepithelial deposition of graft infiltrating mononuclear cells on d90 post transplantation. **b**, **d** Subepithelial deposition and quantification of collagen. Blue bands represent subepithelial collagen deposition, and quantification of collagen blue bands was performed using ImageJ program. “E” and “SE” designate graft epithelial and subepithelial areas respectively. Data are shown as means with SE and representative images of at least two different experiments (*n* = 12) *p < 0.05. Original magnification, × 40

Inflammation-associated injuries, hypoxia, and ischemia during alloimmune reaction are the key leading pathological events that ultimately develop irreversible fibrotic remodeling during the terminal phase of chronic rejection [1]. To demonstrate the progression of pathological remodeling and collagen deposition, we microscopically examined H&E- and trichrome-stained untreated allografts and iPSC-derived MSC-treated allografts on d90 post transplantation. The data revealed that iPSC-derived MSC treatment significantly suppressed subepithelial infiltration of mononuclear cells and thereby facilitated airway epithelial repair at d90 as compared to untreated control allografts, which remain denuded, show significantly huge subepithelial deposition of mononuclear cells, and do not show any associated epithelial repair (Fig. 6a, c). Furthermore, trichrome staining of iPSC-derived MSC-treated allografts demonstrated a significant low subepithelial collagen deposition at d90 compared to untreated control allografts (Fig. 6b, d). This finding might have been related to the fact that the presence of regulatory T cells in graft maintained a favorable microenvironment for graft to revive and avoid inflammation-associated injuries.

## Discussion

The novel regulatory activities of stem cells have been extensively studied in different models of transplantation, which highlighted a crucial immunosuppressive and immune tolerance via the development of regulatory cells, Notch, IDO signaling, TGF-β, PGE2, HLA-G, iNOS, HO-1, galactin-1, and PD-1 expression [52–55]. Solid organ rejection is mediated by “direct” (involving CD8^+^ or CD4^+^ T cells recognizing donor MHC Class I/II molecules) and “indirect” T cell recognition (involving recognition of peptides released by the allogeneic tissue) of alloantigens. In the last couple of years, cell therapy is emerging as an alternative for the drug-based therapies, and among them, MSCs appear as a key choice for the treatment of transplanted patients [5, 56]. MSCs may limit or prevent these outcomes, as they have been shown to suppress the T lymphocyte proliferation induced by alloantigens, including CD4^+^ and CD8^+^ T cells. A particularly interesting application of MSCs is as an adjunct to solid organ transplantation, with a view to inducing allograft tolerance and potentially reducing the incidence of transplant rejection and/or the need for immunosuppressive drugs, in particular calcineurin inhibitors. The latter possibility in itself could be important, given that calcineurin inhibitors have been shown to cause renal failure, hypertension, and hyperglycemia and increase the risk of malignancy [12, 13, 22, 52, 54].

MSCs have the ability to interact with other immune cells, including T cells, regulatory T cells, B cells, dendritic cells, natural killer cells, neutrophil, and macrophages, and thereby check inflammatory phase and repair damaged tissues at the site of inflammation through the release of indoleamine 2, 3-dioxygenase, nitric oxide, TSG-6, IL-10, CCL2, and PGE2 soluble immunosuppressive factors [57]. Immunosuppressive and reparative potential of Tregs has been vital in maintaining immune tolerance and improved long-term transplant outcomes in preclinical and clinical studies [39, 58–63]. Herein, we tested whether and how global immunosuppression by the iPSC-derived human MSC-modulated generation of Tregs during orthotopic mouse trachea transplantation. Our findings demonstrated that iPSC-derived human MSCs and hTSG-6, a protein produced by hMSCs in response to transplant-associated injuries, delayed the onset of acute rejection by augmenting peripheral and intragraft Tregs in a mouse model of orthotopic tracheal transplants. The present study was the “first of its kind” to test the therapeutic efficacy of iPSC-derived MSCs in transplantation, supported the notion that iPSC-derived MSC therapy induces immune tolerance, and thereby supports the reparative phase of microvascular and epithelial repair. Our results successfully demonstrated that iPSC-derived human MSC-mediated immunotherapy increases subepithelial hTSG-6 protein upregulation in mouse tracheal allografts, which is accompanied by a state of immune tolerance for graft reparative cellular activities, reinstates donor-recipient graft microvascular blood flow connections, and, as a result, limits graft-associated injury and progression of collagen deposition during allograft rejection.

In additional studies of cytokine concentrations, iPSC-derived human MSC-mediated immunosuppression led to a significant increase in serum levels of IL-5, IL-10, and IL-15, while reduced levels of IFN-γ, IL-1β, IL-2, IL-23, and IL-6 serum cytokines were observed. Altogether, this anti-inflammatory and immunoregulatory state seems to contribute to an improved immunotolerance, which favors tissue-associated microvascular repair, and oxygenation and blood flow state of transplant. These findings are well supported by earlier preclinical and clinical studies of stem cell therapy, which are well known to induce immune tolerance and delay allograft rejection [12, 13, 20–22, 43, 52, 54, 55, 64, 65]. Furthermore, a number of transplant studies have shown that stem cell therapy stimulates murine Treg cells; stimulates iTregs from conventional T cells; increases FOXP3 gene expression; is able to suppress the proliferation, activation, and differentiation of CD4^+^ T cells and IL-10 secretion; limits Treg conversion to IFN-γ/TNF-α producing T effector cells; and thereby regulates graft versus host disease [66, 67]. Consistent with previous findings, our results also demonstrated that iPSCs-derived MSC-treated allograft expresses high levels of CD4^+^FOXP3 cells compared to low FOXP3 expression in untreated allograft controls. The high levels of CD4^+^FOXP3 regulatory T cell expression and associated regulatory cytokines were closely related with the suppression of acute rejection phase and progression of microvascular repair, and we strongly believe that iPSC-derived MSC-mediated immunosuppression is directly related to the increased infiltration of CD4^+^CD25^+^ FOXP3^+^ cells in the graft area followed by an effective decrease in CD4^+^ effector T cells, which favors graft-specific reparative phase and microvascular-associated graft repair [68] through the upregulation of regulatory cytokines (IL-5, IL-10, and IL-15), while downregulating IFN-γ, IL-1β, IL-2, IL-23, and IL-6 serum cytokines. Furthermore, iPSC-derived MSC-mediated immunosuppression preserved microvascular blow flow, improved tissue oxygenation and airway epithelial repair, and suppressed progression of collagen during rejection. This data indicated that an increase in Tregs and corresponding repair of donor-recipient graft microvasculature in iPSC-derived MSC-treated allografts is highly associated with the transplant epithelial and structural salvage, which is characterized by a drop in subepithelial fibrosis, and ciliated pseudostratified columnar epithelium compared to untreated allografts. Large amount of data from various transplantation models have reported that epithelial cell destruction during ongoing alloimmune inflammation occurs due to several inflammatory mediators, which obstruct regeneration of epithelial and promote fibro-proliferation due to irregular airway tissue repair during rejection phase [1, 7, 39, 69]. These inflammation-associated epithelial injuries have been recognized as a key intermediate pathological event that is ultimately leading to the progression of obliterative airway disease [1, 7, 39, 69].

## Conclusion

iPSC-derived MSC-mediated immunosuppression is sufficient to increase peripheral CD4^+^FOXP3^+^ Tregs, which regulate reparative and anti-inflammatory activities to alloimmune inflammation during rejection. These preclinical findings demonstrate that iPSC-derived MSC-mediated immunosuppression is a key target for facilitating Treg-mediated transplant tolerance. Taken together, these findings highlight the key immunomodulatory potential of novel iPSC-derived MSCs on Treg-mediated immune tolerance, which validate a proof-of-concept that iPSC-derived MSC-mediated immune-suppression is sufficient to establish Treg-mediated immunotolerance during transplantation; further studies are needed to investigate the therapeutic efficacies of Cymerus iPSC-derived MSCs with other existing therapeutic options in the induction of long-term allograft tolerance, and this data might have vital implications for future therapeutic alternatives for lung transplantation.

## Data Availability

The datasets used and/or analyzed during the current study are available from the corresponding author on request.

## Abbreviations

ANOVA: Analysis of variance
BPU: Blood perfusion units
d: Day
FOXP3: Forkhead box P3
FITC: Fluorescein isothiocyanate
iPSC: Induced pluripotent stem cell
MSC: Mesenchymal stem cell
OTT: Orthotopic tracheal transplant
PBS: Phosphate-buffered saline
PFA: Paraformaldehyde
tpO2: Tissue oxygenation
TSG-6: Tumor necrosis factor-stimulated gene 6 protein
Tregs: Regulatory T cells
VEGF: Vascular endothelial growth factor
